# Perennial herb diversity contributes more than annual herb diversity to multifunctionality in dryland ecosystems of North-western China

**DOI:** 10.3389/fpls.2023.1099110

**Published:** 2023-02-15

**Authors:** Hao Guo, Xiao-bing Zhou, Ye Tao, Jin-fei Yin, Lan Zhang, Xing Guo, Chao-hong Liu, Yuan-ming Zhang

**Affiliations:** ^1^ State Key Laboratory of Desert and Oasis Ecology, Xinjiang Institute of Ecology and Geography, Chinese Academy of Sciences, Urumqi, China; ^2^ University of Chinese Academy of Sciences, Beijing, China

**Keywords:** biodiversity, ecosystem multifunctionality, herbs, mass ratio effect, richness effect

## Abstract

**Background:**

Considerable attention has been given to how different aspects of biodiversity sustain ecosystem functions. Herbs are a critical component of the plant community of dryland ecosystems, but the importance of different life form groups of herbs is often overlooked in experiments on biodiversity-ecosystem multifunctionality. Hence, little is known about how the multiple attributes of diversity of different life form groups of herbs affect changes to the multifunctionality of ecosystems.

**Methods:**

We investigated geographic patterns of herb diversity and ecosystem multifunctionality along a precipitation gradient of 2100 km in Northwest China, and assessed the taxonomic, phylogenetic and functional attributes of different life form groups of herbs on the multifunctionality.

**Results:**

We found that subordinate (richness effect) species of annual herbs and dominant (mass ratio effect) species of perennial herbs were crucial for driving multifunctionality. Most importantly, the multiple attributes (taxonomic, phylogenetic and functional) of herb diversity enhanced the multifunctionality. The functional diversity of herbs provided greater explanatory power than did taxonomic and phylogenetic diversity. In addition, the multiple attribute diversity of perennial herbs contributed more than annual herbs to multifunctionality.

**Conclusions:**

Our findings provide insights into previously neglected mechanisms by which the diversity of different life form groups of herbs affect ecosystem multifunctionality. These results provide a comprehensive understanding of the relationship between biodiversity and multifunctionality, and will ultimately contribute to multifunctional conservation and restoration programs in dryland ecosystems.

## 1 Introduction

Biodiversity is a result of the interaction among the species and their environment and ecological processes (Hector and Bagchi, 2007; Manning et al., 2018). Additionally, ecosystem functions refer to the various roles embodied by ecosystems, such as chemical cycling, energy flow and information transfer (Byrnes et al., 2014; Hautier et al., 2018). With species extinctions accelerating globally, there is growing concern that reduced biodiversity affects ecosystem functions (Jing et al., 2015; Delgado-Baquerizo et al., 2016). The multiple dimensions of biodiversity include taxonomic diversity, phylogenetic diversity reflecting evolutionary history, and functional diversity reflecting resource utilization strategies (Willig, 2011; Richter et al., 2021; Suter et al., 2021). In recent decades, ecologists have realized that the traditional sense of taxonomic diversity, i.e. the number of species, ignores the differences in evolutionary history and ecological functions of species, and therefore phylogenetic and functional diversity have received considerable attention (Loewen et al., 2020; Nicholson et al., 2020). Although the multiple attributes of biodiversity are not necessarily correlated (Le Bagousse-Pinguet et al., 2019), an integrated assessment of the relationship between biodiversity and ecosystem multifunctionality in different dimensions is needed. This can help further understanding of the evolutionary history and loss of ecosystem functions due to future species extinctions, and maximize the potential for conservation effectiveness (Gonzalez et al., 2020; Scherer-Lorenzen et al., 2022).

There is growing evidence that the diversity of functional traits in communities is often more important than species richness. This is because higher functional trait diversity is considered a strong environmental filter against gradients in resource use strategies or climatic gradients, for example, specific leaf areas (SLA) (Iknayan et al., 2014; Cadotte et al, 2015; Laughlin et al., 2020). In contrast to species taxonomic diversity, knowledge of functional trait diversity provides researchers with an objective measure of organism roles in ecosystem function through their impact on species growth, reproduction and survival (Cadotte, 2017; Krishnadas et al., 2018). The strength and specific form of the role of species taxonomic diversity concerning ecosystem function is also determined (number of Species) (Díaz et al., 2013). Furthermore, although functional diversity is promising, the number of measurable traits that contain relevance to ecosystem function is often relatively low, and whether specific traits are functionally important, for example for species interactions, is often unclear (Roscher et al., 2011). Assuming that many traits are phylogenetically conserved (Burns and Strauss, 2012; Tucker et al., 2017), phylogenetic diversity can be integrated to account for multiple functional differences between plant species, and thus can be used as a parsimonious and robust indicator of biodiversity. Previous studies have shown that communities with high phylogenetic diversity are more stable and have ecosystems that function with higher productivity and more species at different trophic levels (Cadotte et al., 2015; Flynn et al., 2011; Srivastava et al., 2012). In contrast, low levels of phylogenetic diversity reflect communities that are relatively vulnerable to environmental change, less productive and relatively homogenous in terms of species variety and structure (Cadotte, 2015). Hence, by highlighting the different community components that may influence ecosystem functions, the quantification of multiple attributes of diversity may help to infer and provide additional insights into the mechanisms of action underpinning how biodiversity affects multifunctionality (multifunctionality is the ability of an ecosystem to provide multiple functions and services simultaneously) (Bullock et al., 2011).

Among the most exciting findings in the study of biodiversity and ecosystem multifunctionality over the past decade is that the number of species, regardless of their status, can significantly affect ecosystem functioning (i.e. the cycling of energy, nutrients and organic matter that keeps ecosystems functioning) (Le Bagousse-Pinguet et al., 2019). The implication of this concept (richness effect) is that species loss usually harms ecosystems (Fay et al., 2008; Grime, 1998). Nonetheless, there has been debate about whether the effects of biodiversity on ecosystem functioning primarily reflect the effects of species richness (Moi et al., 2021). However, those studies ignore that even in species-rich vegetation, most of the plant biomass may be found in a few dominant species the characteristics of which can determine the inputs to the primary production of the ecosystem. In other words, much biomass depends on the trait and functional diversity of dominant plants and is insensitive to the relative abundance of subordinate and transitional plants (mass ratio effect) (Ma et al., 2021; Grime, 1998). This means that declines in diversity may be associated with less apparent effects, which may arise through the failure of filters and founder effects (Grime, 1998; Avolio et al., 2019). In particular, we suspect that vegetation dynamics and ecosystem reassembly continue in a context of reduced propagules, leading to a possible progressive loss of function. Under this hypothesis, the importance of plant diversity concerning the deterioration of ecosystem function may derive primarily from the effect on the recruitment of dominant species rather than from any direct effect of richness per se (Wardle et al., 2013; Chaves et al., 2021). Consequently, whether we are considering the taxonomic, functional or phylogenetic dimensions of diversity, the influence of dominant and subordinate plant species on multifunctionality should be more fully understood in conjunction with richness and mass ratio effects.

Dryland is defined as an area with an aridity index (AI) of less than 0.65. Drylands store 20% of the global carbon pool, and their net primary production (NPP) accounts for 30–35% of global NPP (Reynolds et al., 2007), which is closely associated with dryland plants. In Northwest China, arid, semi-arid, and semi-humid arid ecosystems (i.e. drylands) experience a continuous natural vegetation gradient from desert to meadow grassland and account for over 35% of China’s land area (Su et al., 2021). Unfortunately, global climate change, which cause changing amount of precipitation and this cause changing land use and desertification (Dai, 2013; Su et al., 2021). These changes may have significant impacts on biodiversity and associated ecosystem functions. Moreover, as an essential component of dryland ecosystems, herbs (herb refers to plants with underdeveloped xylem in the stem, few lignified cells and weak support force) account for about 67% of the total flora of the Northwest drylands in China (Dang and Pan, 2002; Li et al., 2013; Meng and Zhang, 2013; Meng et al., 2015). Additionally, herbs are not only important indicator species for the resource and environmental status of the region (Luo et al., 2021; Zhou et al, 2020) but also have unique roles and special status in supporting material cycling, maintaining ecosystem functions and coping with climate change in ecologically fragile and sensitive environments (Li et al., 2021; Nie et al., 2019). Herbs include different life form groups (i.e. perennial and annual herbs) (He et al., 2016). Because different life form groups of herbs use environmental resources differently, the factors that lead to changes in ecosystem functions may differ among those groups (Gómez-Aparicio, 2009).

Recent studies of ecosystem multifunctionality in the region have indicated that herb richness is increasing with the increasing amount of annual precipitation through different regions, and drives over 30% of ecosystem function (Hu et al., 2021; Su et al., 2021). Furthermore, there are differences in the effects of the diversity of different types of herbs on ecosystem multifunctionality, which may be due to their different life history strategies indirectly regulating changes in multifunctionality (i.e. herbs of different groups differ markedly in the numbers, phenology and trait characteristics affected by the environment, This also reflects the dynamic balance between resource accessibility and conservative strategies of herbs on the environmental gradient.) (Meng et al., 2015; Wang et al., 2022). Despite this, it is currently unclear which types of herb play a dominant role in influencing multifunctionality. Thus, improving our understanding of the impact of diversity of different herb types on multifunctionality in the Northwest Chinese drylands. It will improve our ability to predict resistance or resilience of the communities. To address these knowledge gaps, we first used multiple attributes of diversity indicators to explain the changes in taxonomic, functional and phylogenetic diversity of total, perennial and annual herbs driving multifunctionality. Naturally, to reveal the influence of dominant species (mass ratio effect) and subordinate species (richness effect) of different groups on multifunctionality, we selected weighted and unweighted indicators of diversity for quantitative analysis. Ultimately, we combined abiotic factors (climate, soil and geography) to holistically assess the extent to which different life form groups of herbs diversity affect multifunctionality at a spatial scale of 2100 km in the drylands of northwest China. The following hypotheses were proposed:

H1: Mass ratio and richness effects are mainly used to elucidate the relationship between the diversity and multifunctionality of dominant and subordinate species. The mass ratio and richness effects of different life form groups of herbs will drive the relationship with multifunctionality, i.e. perennial herbs will be dominated by the mass ratio effect and annual herbs by the richness effect.

H2: The multiple attribute diversity of perennial herbs makes a more significant contribution than does that of annual herbs in explaining multifunctionality.

## 2 Materials and methods

### 2.1 Study site description

This study was conducted along a 2100-km west–east transect over arid and semi-arid regions in northern China, which exhibits a contrasting precipitation gradient from 65 to 443 mm (**Figure S1**). The study area covered six deserts in northern China, namely the Gurbantungut, Badangilin, Ulanbuhe, Kubuchi, Mawusu and Tengri deserts. It also covered a large diversity of vegetation and soil types (Hu et al., 2021). The dominant shrubs were *Haloxylon ammodendron* and *Nitraria tangutorum*, the dominant herbaceous plants were *Agriophyllum squarrosum* and *Stipagrostis pennata*. Our study focused on herbs in the study area, and there was significant variation in herb richness of different life form groups of herbs (2–14 herbs in total per site, average = 6.36; 2–5 perennial herbs per site, average = 3.93; 2–9 annuals per site, average = 4.21). The soil types in the study site were predominantly grey and loess, and the climate was mostly temperate continental (Hu et al., 2020; Su et al., 2021).

### 2.2 Field investigation and sampling

Vegetation surveys were performed during the growth peak of the vegetation season (June–July 2021) according to the local phenology. The field survey was based on an east-west lateral route, with 50 sites set up at 30–50 km intervals along precipitation gradients (all sites were set up in hilly lowlands where the vegetation was in good condition and undisturbed). A 30 × 30 m plot was set up at each site, and five 2 × 2 m herb subplots were set up at equal distances within each plot, using a five-point sampling method (five biological repetitions). Afterwards, recorded all vascular plant species, except woody species found in the aboveground vegetation. Leaf length (LL, cm), leaf width (LW, cm) and plant height (H, cm) were also measured for all species. In addition, five intact leaves were collected from each plant, and leaf area (LA) was measured using a leaf area meter (LI-3100 area meter, LI-COR, Lincoln, USA). After obtaining the leaf dry mass content (LDM), specific leaf area (SLA) was calculated from the LA and LDM. Finally, the herbs surveyed in the subplots were harvested. After being brought back to the laboratory, they were dried in an oven at 60°C for 12 h and their aboveground biomass and leaf chemical characteristics were determined.

A prominent feature of drylands is the ‘fertility island’ effect due to the discontinuous distribution of plants. To avoid a high degree of heterogeneity in soil properties, five cores from 0-10 cm depth were taken at each subplot, and afterwards this five replicates were mixed. Thus we had five aggregated samples from each plots. When brought back to the laboratory, five cores were mixed to create one replicate. This procedure was replicated five times within the five subplots to generate five biological replicates. Finally, the collected soil samples were air-dried in preparation for soil property analysis.

### 2.3 Plant and soil property measurements

For each plant individuals in each subplot, a total carbon analyzer was used to determine the total carbon content of the plant leaves. Leaf total nitrogen and total phosphorus contents were determined using a continuous-flow ion auto-analyzer (Auto-Analyzer 3, Germany) (Lambers, 2021).

The soil organic carbon (SOC) content was determined using the dichromate oxidation method (Urbansky, 2001). Soil total nitrogen (TN), inorganic nitrogen (IN), organic nitrogen (ON) and alkali-hydrolyzable nitrogen (AN) contents were determined using a continuous-flow ion auto-analyser (Shamrikova et al., 2022). Soil total phosphorus (TP) content was measured with the HClO_4_-H_2_SO_4_ ammonium molybdate-ascorbic acid method (Lambers, 2021). The molybdenum counterstain method was used to determine the soil content of activated phosphorus (AVP) and inorganic phosphorus (IP) (Gilbert et al., 2009). In addition, soil pH was measured using a pH meter (FiveEasy FE20, Switzerland) placed in a 1:2.5 (v/v) soil/water extract. Soil water content (SWC) was calculated from soil weight, indicated as soil moisture as a percentage of dry soil mass (Ritchie, 1981; Luo et al., 2021).

### 2.4 Multiple attribute indicators of biodiversity

Taxonomic diversity is the most intuitive and commonly used measure of biodiversity, and is determined mainly by the number of species (Wang et al., 2021). We used species richness to indicate taxonomic diversity.

Functional diversity in this study included FDis (functional dispersion, the mean distance in multidimensional trait space of individual species to the centroid of all species), w.FDis (weighted by species abundance functional dispersion) and CWM (community-weighted mean) (Lavorel et al., 2008). For the calculation of FDis, leaf length, leaf width and plant height were used as functional traits; they all reflect light retention and water tolerance (Westoby et al., 2002). In addition, for the calculation of CWM, we chose SLA, which relates to the relative growth rate and nutrient acquisition and utilization of the plant (Wright et al., 2004).

Phylogenetic diversity is relevant to species evolution. We determined the phylogenetic diversity indices using the ‘picante’ package (1.8.2), including MNTD (mean nearest taxon distance) and w.MNTD (mean nearest taxon distance weighted by species abundance) (Webb et al., 2002; Kembel et al., 2010).

Richness effects include species richness, FDis and MNTD. Quality ratio effects include weighted w.FDis, w.MNTD and CWM.SLA.

### 2.5 Ecosystem multifunctionality

Multifunctionality is a composite measure of an ecosystem’s ability to maintain multiple functions simultaneously (Le Bagousse-Pinguet et al., 2019). Twelve functions, grouped into three functional categories (C-cycle, N-cycle and P-cycle) were used for calculations (**Table S1**). These variables form a good proxy for the biological productivity and nutrient cycling of ecosystem functions. We used multiple thresholds to assess the effects of plant diversity on multifunctionality. Multi-threshold-based calculations provide a powerful and flexible method for assessing multifunctionality, which enables the number of well-performing functions to be captured and the nature and extent of multifunctionality in an ecosystem to be assessed quantitatively, even when there are trade-offs between those functions.

### 2.6 Abiotic variables

In terms of climate, mean annual precipitation (MAP) and temperature (MAT) were obtained from the World Climate Database (www.worldclim.org) at a resolution of 30 arc minutes per sample site. The aridity level of each site was calculated as an AI (ratio of precipitation to potential evapotranspiration), which obtained from the Global Drought Index and Potential Evapotranspiration Climate Database (https://cgiarcsi.community/) (Hu et al., 2021). For soil variables, we used soil water content and pH. In addition, longitude, latitude and elevation were recorded at all plots and applied as spatial variables. These indicators play a crucial role in the availability of dryland ecosystem functions. For example, MAP and MAT have important effects on vegetation and soil properties in drylands, which in turn directly or indirectly regulate multifunctionality.

### 2.7 Statistical analysis

We assessed the impact of different types of herb diversity on the multifunctionality of drylands. First, all variables were z-cores normalized. We calculated the correlations between the multifunctionality indicators. Afterwards, among the plant diversity and environmental indicators. When a correlation coefficient above 0.6 for a pair of candidate variables was observed, one variable was eliminated to avoid high autocorrelation between variables. The correlation between plant diversity indicators was not significant. For indicators related to environmental and multifunctionality, we excluded latitude, drought index (AI), inorganic phosphorus (IP), alkaline nitrogen (AN) and organic nitrogen (ON) (**Table S1**, **S2**). Because of the high correlation coefficients.

In terms of the relationship between diversity (taxonomic, functional and phylogenetic diversity) and multifunctionality in different life form groups of herbs, we used the ‘multifunc’ package to calculate slopes (Byrnes et al., 2014). The slopes were used to fit a linear mixed model to estimate the linear relationships, which was replicated at thresholds from 0 to 100% (at 1% intervals). The linear relationship predicted the number of functions that performed at or above the threshold by the function used to indicate diversity. This approach is used to investigate how the shape of the fitted curve changes at different thresholds and to determine the interval from the minimum threshold to the maximum threshold variation (Byrnes et al., 2014). The minimum threshold, Tmin, is the minimum threshold at which the slope is statistically significantly greater or statistically significantly less than 0 when diversity affects multifunctionality. The maximum threshold, Tmax, is the threshold at which the effect of diversity on multifunctionality becomes insignificant as the threshold increases, i.e. the maximum threshold at which the slope is not 0. We took the same approach for climate (MAT and MAP), soil (SWC and pH) and geography (longitude and elevation) variables.

To assess the effect of mass ratio and richness effects on multifunctionality, comparisons were made between the absolute values of their standardized regression coefficients and the sum of the standardized regression coefficients of all variables (Le Bagousse-Pinguet et al., 2019). The significance was represented as the percentage of variance explained. This is similar to a partitioned analysis of variance. The net effect of diversity was calculated as the sum of the standardized regression coefficients for all biodiversity indicators during the model selection process. We then examined the following identifiable variance scores: climate (MAT and MAP), soil (SWC and pH) and geography (longitude and elevation) and each of the biodiversity indicators.

Finally, multiple regression models were used to assess the relationships between different life form groups of herbs diversity and multifunctionality. We fitted the models to all predictor variables using the maximum likelihood (ML) method within the ‘MuMin’ package (Le Bagousse-Pinguet et al., 2019; Wang et al., 2021). The models were also subjected to a selection procedure based on ΔAICc < 2 using AICc (Akaike Information Criteria) to determine the best predictor variables for ecosystem functioning. All statistical analyses and visualizations were conducted in R (R Development Core Team 2017).

## 3 Results

### 3.1 The relationship between diversity and multifunctionality of different life form groups of herbs

The 95% confidence intervals around the estimated slopes reveal whether the estimates overlap 0, giving a test of the threshold values at which diversity has no effect on multifunctionality. Across the transect, according to the results of the multiple threshold analysis, the species richness, FDis and CWM.SLA of all herbs together were positively correlated with Tmin 45%–Tmax 95%, Tmin 46%–Tmax 96% and Tmin 57%–Tmax 98% of the multi-thresholds of ecosystem functioning (**Figure S2**). Perennial herbs species richness, FDis, w.FDis and CWM.SLA were correlated with Tmin 46%–Tmax96%, Tmin44%–Tmax92%, Tmin41%–Tmax92% and Tmin56%–Tmax99% of multifunctionality (**Figure S3**). The annual herb diversity indicators were more correlated with multifunctionality than were the perennial herbs, with species richness, FDis and CWM.SLA explaining Tmin41%–Tmax92%, Tmin43%–Tmax96%, and Tmin56%–Tmax 96% of the multi-thresholds (**Figure S4**). We also found that the MNTD and w.MNTD of herbs did not correlate with the multifunctionality of the whole threshold.

### 3.2 Explanation of the mass ratio and richness effects of different life form groups of herbs on multifunctionality

**Figure 1:**
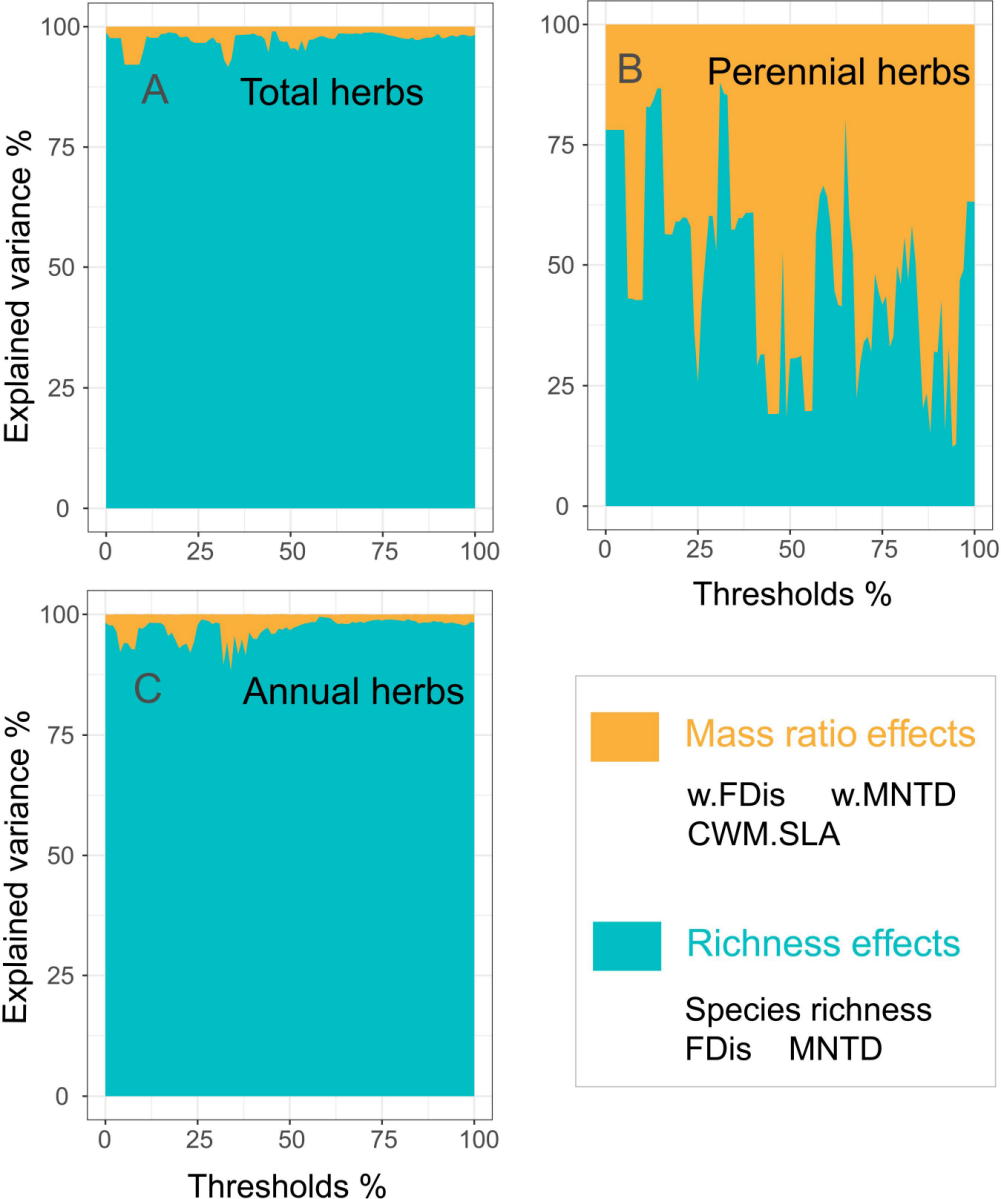
Mass ratio and richness effects of herbs were significant for multifunctionality. The significance of the predictor variables is expressed as the percentage of variation they explain and is based on the absolute value of their standardized regression coefficients (**A**, total herbs; **B**, perennial herbs; **C**, annuals herbs). Abbreviaions are as in **Table S2**.

Biodiversity effects caused by dominant and subordinate species may explain the relationship with ecosystem functioning in greater depth. Our study quantified mass ratio and richness effects for different types of herbs within an overall threshold interval. The richness effects of total and annual herbs (both 97%) better explained multifunctionality than did the mass ratio effect (**Figures 1A, C**). In contrast, the mass ratio effect for perennial herbs contributed more to multifunctionality (53%) than to the richness effect (47%) (**Figure 1B**). This suggests that the richness effect due to subordinate species of annual herbs is the best predictor of multifunctionality, while the dominant species of perennial herbs play a significant role in the impact of multifunctionality in the northwest drylands (**Figure 1**).

**Figure 2:**
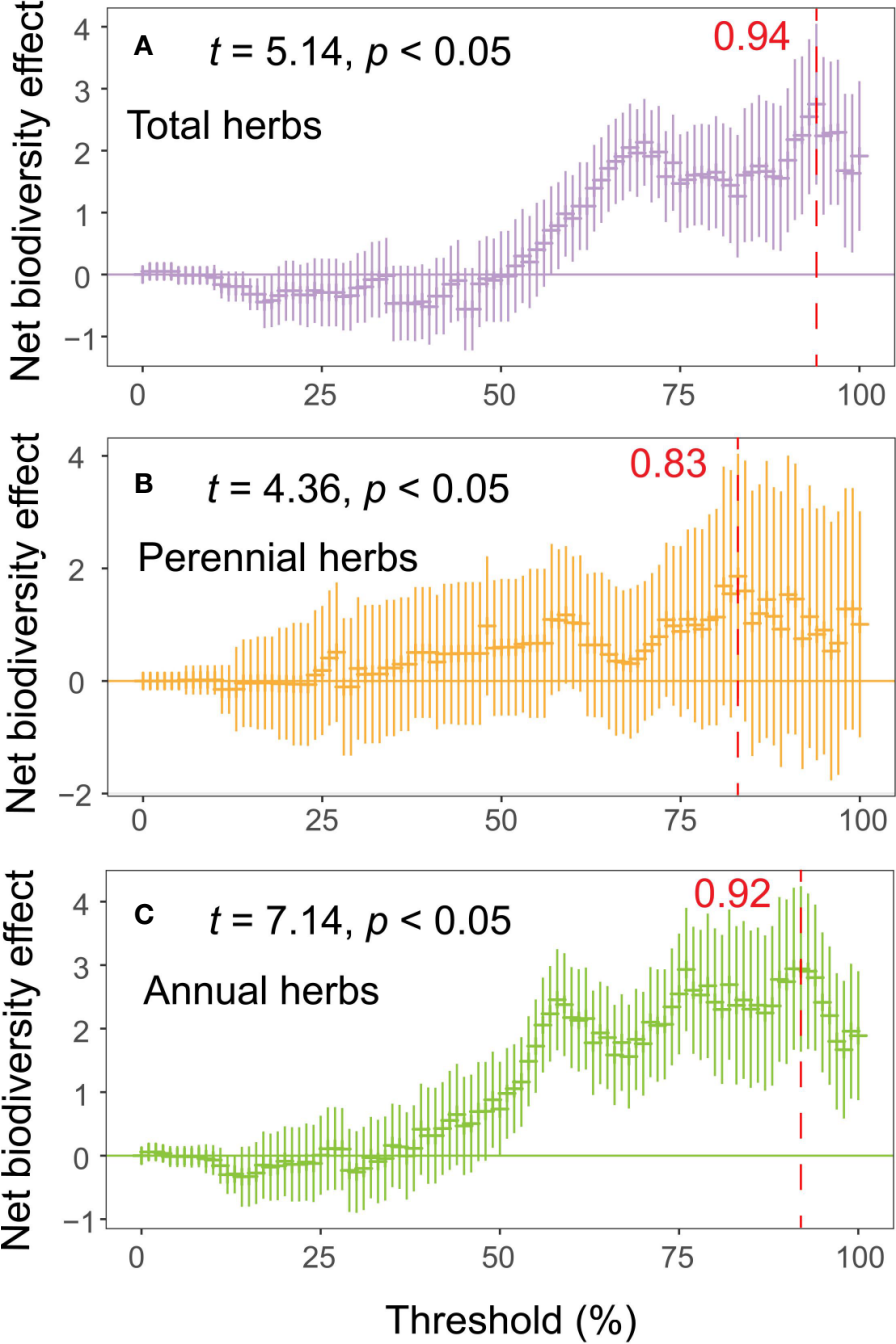
Net effect of biodiversity attributes multifunctionality. The net effect is calculated as the sum of the standardized regression coefficients for all biodiversity indicators selected in the model selection process (**A**, total herbs; **B**, perennial herbs; **C**, annuals herbs).

With increasing thresholds, the net effect and multifunctional relevance of the different life form groups of herbs gradually increased (**Figure 2**). The peak was reached at 94, 83 and 92% of the threshold for total, perennial and annual herbs (**Figure 2**), despite the weaker net effect of diversity on multifunctionality at lower thresholds. However, the multiple attributes of overall diversity enhanced the multifunctionality of the ecosystem.

### 3.3 The contribution of herb diversity attributes to multifunctionality

**Figure 3:**
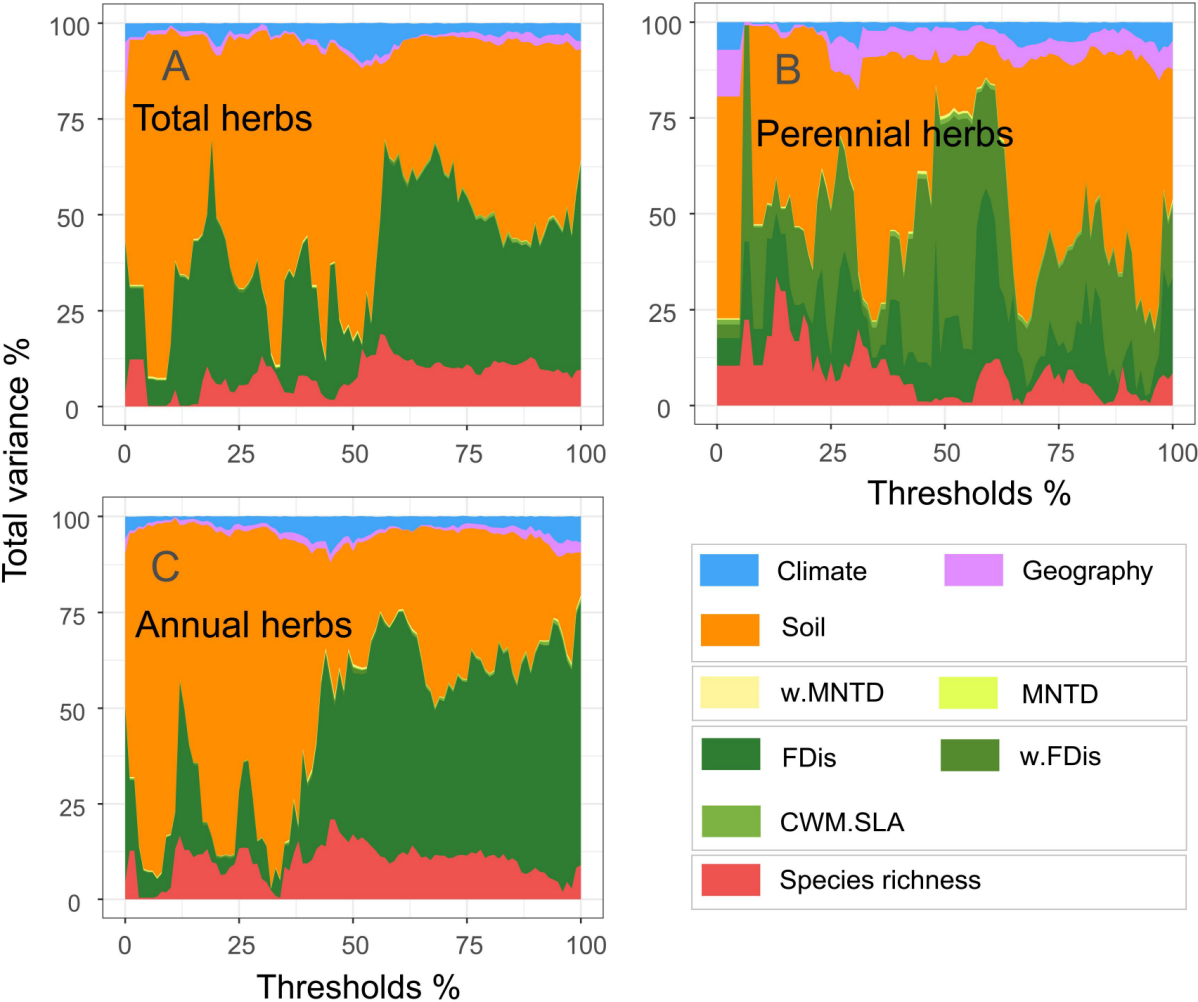
Relative importance of climate, geography, soil variables and biodiversity indicators on multifunctionality (**A**, total herbs; **B**, perennial herbs; **C**, annuals herbs).

When climate, soil and geographical variables were considered, total, perennial and annual herb diversity explained 41, 49% and 47% of the multifunctional variation (**Figure 3**). Of these, perennial herbs contributed more to multifunctionality than did annual herbs. In addition, functional diversity had the most substantial impact on multifunctionality among the multiple attributes of diversity. Functional diversity explained 33, 40 and 36% of the variation (**Figure 3**). Although taxonomic and phylogenetic diversity explained minor multifunctionality, they also contributed to ecosystem function. These results highlight the need to combine specific combinations of diversity attributes to understand ecosystem multifunctionality.

**Figure 4:**
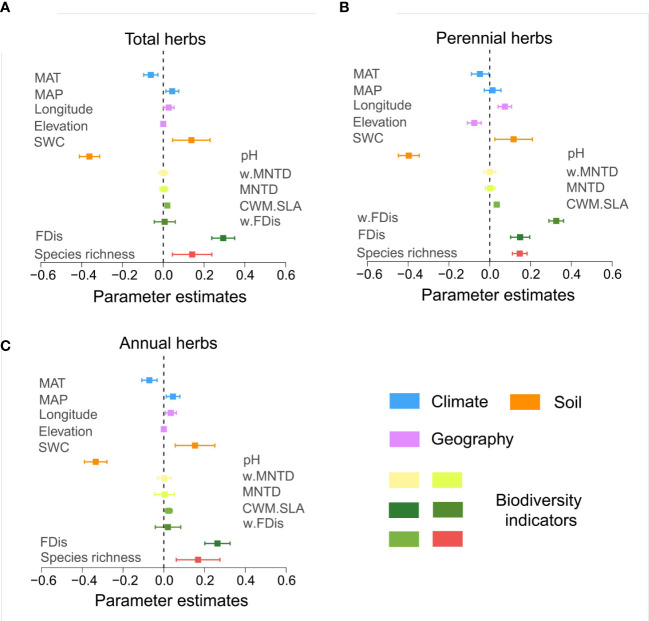
Standardized regression coefficients and associated 95% confidence intervals for the multifunctional model predictor variables. Standardized regression coefficients were derived from the model averaging procedure and averaged over the threshold range (0-100%). Confidence intervals that do not cross the zero line indicate that the predictor variables considered are associated with a statistically significant (*p* < 0.05). See **Table S3** for the mean thresholds of the standardized regression coefficients for each predictor variable. (**A**, total herbs; **B**, perennial herbs; **C**, annuals herbs).

The predictive variables analysis, species richness, FDis and CWM.SLA of different life form groups of herbs were positively correlated with multifunctionality (*p*<0.05). In contrast, MNTD and w.MNTD were not correlated with multifunctionality (*p*>0.05) (**Figure 4**). Annual herbs, of w.FDis also behaved similarly (**Figures 4A, C**). This may be due to the different survival strategies developed by different life form groups of herbs to adapt to the dryland environment, leading to inconsistent patterns of effects on multifunctionality.

## 4 Discussion

Understanding the relationship between herb diversity and multifunctionality in different life form groups of herbs provides a better understanding of the complex influence of herbs on dryland ecosystem function. Our study synthesized the effects of the multiple biodiversity attributes (taxonomic, phylogenetic and functional diversity) of herbs on multifunctionality in the drylands of northwest China. In larger-scale geographic and climatic environments, perennial and annual herbs drive changes in multifunctionality in terms of mass ratio and richness effects. Furthermore, perennial herbs diversity explains multifunctionality better than annual herbs. Such results emphasize that an integrated consideration of multiple attributes is a critical step towards understanding the potential mechanisms of multifunctionality. Comprehensive studies of different life form groups of herbs provide additional evidence for the multifunctionality of dryland ecosystems.

### 4.1 Mass ratio effects and identification of the richness effects of ecosystem multifunctionality

The simultaneous effects of multiple biodiversity attributes on multifunctionality emphasize the need to move from a single taxonomy to a more multidimensional biodiversity perspective, which is essential for restoring and managing dryland ecosystems. Considering different life form groups of herbs also provides insight into the complex effects of diversity attributes on ecosystem function. Our results suggest that herb diversity in terms of multiple attributes significantly affects the number of functions at the 41–99% threshold (weighted and unweighted indicators were considered). Some studies have shown mass ratio and richness effects of different life form groups of herbs on ecosystem functioning, which is consistent with our results (Bhattarai and Vetaas, 2003; Wang et al., 2021). As hypothesized, the effect of total and annual herb diversity on multifunctionality was mainly driven by richness effects (subordinate species). This may be because spring snowmelt stimulates more annual herbs to simultaneously emerge in large numbers of subordinate species, thus maximizing multifunctionality. In other words, probably the complementarity of ecological niches strongly drove the relationship between annuals and multifunctionality (Stehli et al., 1969). Because subordinate plants will exhibit a high degree of fidelity of association with a particular vegetation groups, i.e. such plants are smaller in stature, make rational use of resources to a more limited extent, and tend to occupy microhabitats defined by associated dominant species and phenology (Grime, 1998; Soliveres et al., 2014). This is also consistent with the general pattern of species distinctiveness in the region (Wang et al., 2021).

Mass ratio effects (dominant species) are critical in driving the multifunctional impact of perennial herbs diversity (Valencia et al., 2015). There is evidence that dominant plants recur in specific vegetation groups, are relatively large individuals, and exhibit a wide range of resource use and, as individual species, an enormous contribution to biomass (selection effect) (Grime, 1998). This conclusion agrees with our results. For perennial herbs, which are less influenced by the environment, their nutrient cycling and phenotypic plasticity are more stable than are those of annuals (Hu et al., 2022). The mass ratio effect was, therefore, more strongly correlated with multifunctionality. Our results extend the study of richness and mass ratio effects in dryland herbs. Although there are differences in how different life form groups of herbs influence ecosystem function, our results reinforce the idea that dryland perennial and annual herbs drive ecosystem multifunctionality with different survival strategies (Hu et al., 2021).

### 4.2 The diverse biodiversity attributes of different life form groups of herbs enhance ecosystem multifunctionality

When multiple biodiversity attributes are considered simultaneously, biodiversity is generally observed to positively affect multifunctionality (Cadotte et al., 2011). The net effect of herb diversity within different life form groups were positively correlated with multifunctionality. This positive relationship may depend on the particular groups of dryland ecosystem function (Bond and Chase, 2002; Wang and Loreau, 2016). A recent study has demonstrated that the net effect of herbs were positively correlated with multifunctionality in the deserts of the Abbey Lake region of China (Wang et al., 2021). This finding is consistent with our own. We speculate that herbs consisting of different herb groups are more likely to contain ecotone broad competitors (Isbell et al., 2018). Positive relationships occur when competitors contribute more to ecosystem functions of interest (e.g. C, N and P cycling) (Jiang et al., 2008). In contrast to our results, in a global dryland study the impact of multiple biodiversity attributes on multifunctionality was relatively weak (Le Bagousse-Pinguet et al., 2019). This weaker relationship may be caused by differences in functional and biodiversity attributes associated with biogeochemical cycling. This evidence suggests that factors affecting multifunctionality depend not only on the combination of functions associated with ecosystems but also on the characteristics of the assessed biodiversity attributes.

Interestingly, in our study, the net effect of total and annual herb diversity on ecosystem multifunctionality was higher than the effect of perennial herb diversity. This may be related to the life history strategies of annual herbs. Small amounts of precipitation can rapidly enhance species abundance (Schwinning and Sala, 2004; Levine and HilleRisLambers, 2009). Thus, higher abundance enhances the net effect of annual herbs overall. In contrast, although perennial herbs drive dryland ecosystem multifunctionality with a mass ratio effect, their relatively low abundance may be the main reason for this difference. This may be the result of different strategies for resource accessibility and conservatism across the environmental gradient for different life form groups of herbs. Similar to our findings, Isbell et al. (2018) found that increasing the number of species enhanced ecosystem function in an experiment on grassland plant diversity. This implies that ignoring the effects of various attributes encompassed by biodiversity measures, such as richness and mass ratio effects, may affect our ability to predict the impact of biodiversity on ecosystem functioning in drylands.

### 4.3 Functional diversity is the primary variable driving ecosystem function, and perennial herbs better explain dryland multifunctionality than do annuals

Our ecosystem multifunctionality results track that have previously found multiple attribute diversity. Previous studies and the present study found that functional diversity of herbs has a stronger relationship with multifunctionality than taxonomic and phylogenetic diversity. (Zuppinger-Dingley et al., 2014; Zhu et al., 2016; Santala et al., 2022). In line with this, previous studies have defined biodiversity as a variety of functional traits in communities or ecosystems rather than the number of species (Reiss et al., 2009; Aubree et al., 2020). This trend is underpinned by growing evidence that taxonomic richness typically has only a weak impact on ecosystem function. Particularly at relatively large geographical scales, functional traits are increasingly considered to be a more appropriate biodiversity indicator. Furthermore, in our results, phylogenetic diversity explains much less of the multifunctionality than does taxonomic and functional diversity. This is not surprising in a study of dryland ecosystems. Environmental constraints and the large number of emergent annual herbs that have difficulty tolerating environments of high-intensity droughts result in a high degree of relatedness between individual plants (species redundancy) (Boulangeat et al., 2012; Schellenberger Costa et al., 2017). This was also verified in our previous survey of plants, with annual herbs being dominated by Asteraceae. For perennial herbs, although more tolerant of extreme drought, it may be that the lower level of species and trait variability encompassed by phylogenetic diversity does not make them a better predictor of ecosystem function than does taxonomic and functional diversity.

As predicted by our second hypothesis, multiple attribute diversity in perennial herbs explained more ecosystem multifunctionality than did multiple attribute diversity in annual herbs. We found that the FDis of all herbs and the FDis of annual herbs were the main variables explaining multifunctionality. There was evidence that communities composed of species with similar trait values would have lower FDis, while communities composed of species with divergent traits would have higher FDis (Villéger et al., 2008; Laliberté and Legendre, 2010). Our results may be explained by higher species richness in annual herbs, resulting in similar patterns in all herbs and annual herbs. In addition, annual herbs are subject to environmental filtering and dispersal constraints, resulting in stronger ecological niche zonation. As a result, species composition produces larger variation in traits (e.g. plant height) (Gross et al., 2017). Perennial herbs are highly adaptable to their environment. Although they are also subject to environmental filtering and dispersal limitations, different species have developed similar drought-tolerant traits throughout long-term evolution, resulting in functional redundancy. This functional redundancy may limit the survival strategies of plants and thus reduce the effects of FDis on multifunctionality (Zheng et al., 2022). Interestingly, perennial herbs of w.FDis significantly influenced multifunctionality. Although there is an effect of species redundancy, the traits of dominant species may be more critical. Strong experimental evidence supports the hypothesis that dominant plant traits strongly influence ecosystem function. In a comparative study of the resistance and resilience of herbs to drought, late frost and fire (MacGillivray et al., 1995), multiple ecosystem functions were found to be strongly correlated with the functional traits of the dominant contributors. Moreover, functional differences between co-occurring dominant species can profoundly impact ecosystems in terms of maintenance of productivity. We also noted that the CWM.SLA of different life form groups of herbs also positively affected multifunctionality. Although weakly explained, it represents a filter for plants along a resource use strategy gradient or a climate gradient (Hart and Chen, 2008; Reich et al., 2012).

## 5 Conclusions

This study provides empirical evidence that the richness effect of annual herbs and the mass ratio effect of perennial herbs are essential factors driving multifunctionality in natural dryland ecosystems. Our findings also suggest that multiple biodiversity attributes positively affect multifunctionality. On a relatively large geographical scale, functional diversity was the best indicator for explaining multifunctionality, and the diversity of perennial herbs explained more multifunctional variation than did that of annual herbs. Our results further emphasize the need to consider multiple attributes of diversity (exceptionally functional diversity) to understand the relationship between biodiversity and ecosystem multifunctionality. Moreover, the importance of different life form groups of herbs in sustaining multifunctional change cannot be overlooked when analyzing mechanisms. In an era of a biodiversity crisis, our findings provide an ecological perspective for maintaining biodiversity and optimizing ecological restoration processes, and contributing to improved management and policy action in drylands.

## Data availability statement

The raw data supporting the conclusions of this article will be made available by the authors, without undue reservation.

## Author contributions

X-bZ and Y-mZ conceived the idea of this study and designed the experiments; field sampling was conducted by HG, YT, J-fY, LZ, and XG; laboratory analyses were done by C-hL; bioinformatics analyses were done by HG; the manuscript was written by HG. All authors contributed to the article and approved the submitted version.
