# Supplementary material for: Perennial herb diversity contributes more than annual herb diversity to multifunctionality in dryland ecosystems of North-western China
Source: Front Plant Sci. 2023 Feb 15;14:1099110. doi: 10.3389/fpls.2023.1099110 (PMC9986965; doi:10.3389/fpls.2023.1099110)
Supplement: Supplementary file 1 [file DataSheet_1.docx]

**Figure S1.** Study area and field sampling sites.





**Figure S2.** The relationship between the diversity of multiple attributes and the multifunctionality of total herbs. Tmin/max = the lowest or highest threshold where biodiversity affects multifunctionality significantly.





**Figure S3.** The relationship between the diversity of multiple attributes and the multifunctionality of perennial herbs. Tmin/max = the lowest or highest threshold where biodiversity affects multifunctionality significantly.

**

**

**Figure S4.** The relationship between the diversity of multiple attributes and the multifunctionality of annual herbs. Tmin/max = the lowest or highest threshold where biodiversity affects multifunctionality significantly.

**

**

**Table S1**. Correlation matrix (Spearman's) between multifunctional indicators of the ecosystem. Highly correlated variables (r > 0.6) are shaded (red). SOC: soil organic carbon content; CWM.C: community-weighted mean of leaf carbon content (herbs); AGB: aboveground biomass; ON: soil organic nitrogen; IN: soil sinorganic nitrogen; TN: soil total nitrogen; AN: soil alkaline nitrogen; CWM.N: community weighted mean of leaf nitrogen content (herbs); TP: soil total phosphorus; IP: soil inorganic phosphorus; CWM.P: community weighted mean of leaf phosphorus content (herbs); AVP: soil activated phosphorus.

| Factors | SOC | CWM.C | AGB | ON | IN | TN | CWM.N | TP | IP | CWM.P | AVP | AN |
| --- | --- | --- | --- | --- | --- | --- | --- | --- | --- | --- | --- | --- |
| TC | 1 |  |  |  |  |  |  |  |  |  |  |  |
| CWM.C | 0.4112 | 1 |  |  |  |  |  |  |  |  |  |  |
| AGB | 0.4374 | 0.1436 | 1 |  |  |  |  |  |  |  |  |  |
| ON | -0.1947 | -0.1973 | -0.1048 | 1 |  |  |  |  |  |  |  |  |
| IN | -0.2639 | -0.2235 | -0.1416 | -0.0900 | 1 |  |  |  |  |  |  |  |
| TN | 0.2843 | -0.0488 | 0.3702 | 0.7293 | -0.1053 | 1 |  |  |  |  |  |  |
| CWM.N | -0.0600 | -0.1990 | 0.1938 | 0.0455 | 0.1878 | 0.0409 | 1 |  |  |  |  |  |
| TP | -0.2884 | -0.3601 | -0.1889 | 0.2121 | -0.0879 | -0.0373 | 0.1388 | 1 |  |  |  |  |
| IP | -0.0148 | -0.1364 | -0.2641 | 0.0046 | -0.0713 | -0.1946 | 0.2884 | 0.9426 | 1 |  |  |  |
| CWM.P | 0.3206 | 0.5317 | 0.2037 | -0.0084 | -0.1563 | 0.3285 | 0.3926 | -0.1502 | -0.1502 | 1 |  |  |
| AVP | -0.0349 | 0.0077 | -0.1987 | 0.2766 | -0.1247 | 0.3176 | 0.2263 | 0.0074 | 0.0074 | 0.3731 | 1 |  |
| AN | -0.0122 | 0.1860 | 0.1287 | 0.0162 | 0.0311 | 0.6192 | -0.0015 | -0.1095 | -0.1695 | -0.0895 | -0.0557 | 1 |

**Table S2**. Correlation matrix (Spearman's) of biotic and abiotic variables. Highly correlated variables (r > 0.6) are shaded (red). MAP: mean annual precipitation; MAT: mean annual temperature; SWC: soil water Content; SR: Species richness; w.FDIS and FDIS: abundance-weighted and non-weighted functional dispersion; w.MNTD and MNTD: weighted and non-weighted Mean Nearest Taxon Distance. CWM.SLA: community-weighted mean of specific leaf area; AI: drought index.

|  | Factors | MAT | MAP | Latitude | Longitude | Elevation | SWC | pH | SR | FDis | MNTD | w.FDis | w.MN  TD | CWM.  SLA | AI |
| --- | --- | --- | --- | --- | --- | --- | --- | --- | --- | --- | --- | --- | --- | --- | --- |
| Total herbs | MAT | 1 |  |  |  |  |  |  |  |  |  |  |  |  |  |
|  | MAP | -0.5523 | 1 |  |  |  |  |  |  |  |  |  |  |  |  |
|  | Latitude | -0.0743 | -0.3814 | 1 |  |  |  |  |  |  |  |  |  |  |  |
|  | Longitude | -0.0641 | 0.4575 | -0.5025 | 1 |  |  |  |  |  |  |  |  |  |  |
|  | Elevation | -0.2347 | 0.0160 | -0.4580 | 0.5588 | 1 |  |  |  |  |  |  |  |  |  |
|  | SWC | -0.0073 | 0.0998 | -0.4885 | 0.3853 | 0.4252 | 1 |  |  |  |  |  |  |  |  |
|  | pH | 0.3491 | -0.5174 | 0.6014 | -0.5733 | -0.4599 | -0.2520 | 1 |  |  |  |  |  |  |  |
|  | SR | -0.2624 | 0.5324 | -0.4078 | 0.4438 | 0.2109 | 0.2477 | -0.3229 | 1 |  |  |  |  |  |  |
|  | FDis | -0.1915 | 0.4736 | -0.2498 | 0.3458 | 0.1479 | -0.0201 | -0.3468 | 0.4197 | 1 |  |  |  |  |  |
|  | MNTD | -0.1119 | -0.0951 | 0.0559 | 0.0326 | 0.0627 | -0.0862 | 0.0087 | -0.2159 | 0.1935 | 1 |  |  |  |  |
|  | w.FDis | -0.0926 | 0.0657 | 0.2573 | -0.2262 | -0.2114 | -0.1341 | 0.1592 | -0.1342 | 0.0845 | -0.0534 | 1 |  |  |  |
|  | w.MNTD | -0.0477 | -0.2090 | 0.1381 | -0.0578 | 0.0265 | -0.1450 | 0.0844 | -0.2673 | 0.2162 | 0.4069 | -0.0118 | 1 |  |  |
|  | CWM.SLA | 0.0056 | 0.5380 | -0.5531 | 0.4113 | 0.3148 | 0.2683 | -0.5299 | 0.4615 | 0.5574 | -0.1619 | -0.0812 | -0.2438 | 1 |  |
|  | AI | -0.5119 | 0.9949 | -0.3505 | 0.4296 | 0.0334 | 0.6092 | -0.5162 | 0.5130 | 0.4595 | -0.0828 | 0.0815 | -0.2033 | 0.5048 | 1 |
| Perennial herbs | MAT | 1 |  |  |  |  |  |  |  |  |  |  |  |  |  |
|  | MAP | -0.5700 | 1 |  |  |  |  |  |  |  |  |  |  |  |  |
|  | Latitude | 0.0352 | 0.6707 | 1 |  |  |  |  |  |  |  |  |  |  |  |
|  | Longitude | -0.2032 | -0.5355 | -0.4976 | 1 |  |  |  |  |  |  |  |  |  |  |
|  | Elevation | -0.0779 | -0.2835 | 0.0685 | -0.5517 | 1 |  |  |  |  |  |  |  |  |  |
|  | SWC | 0.0681 | 0.0172 | -0.3466 | 0.1487 | 0.2348 | 1 |  |  |  |  |  |  |  |  |
|  | pH | 0.5743 | -0.4537 | 0.5466 | -0.5248 | -0.0187 | -0.0342 | 1 |  |  |  |  |  |  |  |
|  | SR | -0.2287 | 0.4769 | -0.4442 | 0.3584 | -0.1440 | 0.3705 | -0.1931 | 1 |  |  |  |  |  |  |
|  | FDis | -0.4759 | 0.4514 | 0.0460 | 0.2355 | -0.1528 | -0.2137 | -0.3828 | 0.2679 | 1 |  |  |  |  |  |
|  | MNTD | 0.0020 | -0.2497 | 0.3714 | -0.1491 | -0.0726 | -0.2692 | 0.1217 | -0.4382 | -0.0355 | 1 |  |  |  |  |
|  | w.FDis | -0.5736 | 0.5220 | -0.3074 | 0.4991 | -0.1988 | 0.2507 | -0.4466 | 0.4963 | 0.4418 | -0.1551 | 1 |  |  |  |
|  | w.MNTD | 0.1411 | -0.3470 | 0.4440 | -0.2131 | -0.0800 | -0.3694 | 0.2062 | -0.5140 | 0.0976 | 0.4561 | -0.2302 | 1 |  |  |
|  | CWM.SLA | -0.3165 | 0.5290 | -0.4632 | 0.4693 | -0.2040 | 0.2273 | -0.4897 | 0.4280 | 0.5007 | -0.4535 | 0.5062 | -0.5057 | 1 |  |
|  | AI | 0.4072 | 0.7150 | 0.4056 | -0.4345 | 0.1528 | 0.4486 | 0.5638 | -0.3277 | -0.0923 | 0.1630 | -0.4660 | 0.4325 | -0.5249 | 1 |
| Annual herbs | MAT | 1 |  |  |  |  |  |  |  |  |  |  |  |  |  |
|  | MAP | -0.5792 | 1 |  |  |  |  |  |  |  |  |  |  |  |  |
|  | Latitude | -0.0297 | -0.3762 | 1 |  |  |  |  |  |  |  |  |  |  |  |
|  | Longitude | -0.1180 | 0.4540 | -0.4909 | 1 |  |  |  |  |  |  |  |  |  |  |
|  | Elevation | -0.2165 | 0.0536 | -0.4723 | 0.6331 | 1 |  |  |  |  |  |  |  |  |  |
|  | SWC | -0.0641 | 0.1024 | -0.4467 | 0.3892 | 0.4233 | 1 |  |  |  |  |  |  |  |  |
|  | pH | 0.3978 | -0.4250 | 0.5731 | -0.5592 | -0.4774 | -0.1954 | 1 |  |  |  |  |  |  |  |
|  | SR | -0.2543 | 0.5013 | -0.4625 | 0.4604 | 0.3160 | 0.3637 | -0.3587 | 1 |  |  |  |  |  |  |
|  | FDis | -0.2304 | 0.5253 | -0.2978 | 0.3977 | 0.1850 | -0.0338 | -0.4058 | 0.4692 | 1 |  |  |  |  |  |
|  | MNTD | -0.1599 | 0.0071 | 0.0571 | 0.0774 | -0.0563 | -0.1893 | 0.0219 | -0.0943 | 0.1963 | 1 |  |  |  |  |
|  | w.FDis | -0.1065 | 0.0717 | 0.2641 | -0.2293 | -0.2092 | -0.1383 | 0.1547 | -0.1495 | 0.0772 | -0.0463 | 1 |  |  |  |
|  | w.MNTD | -0.1475 | -0.1355 | 0.1545 | -0.0473 | -0.0447 | -0.2415 | 0.0974 | -0.1848 | 0.1670 | 0.3161 | -0.0073 | 1 |  |  |
|  | CWM.SLA | -0.0576 | 0.5472 | 0.6489 | 0.3119 | 0.4028 | 0.2543 | -0.5424 | 0.4713 | 0.5147 | -0.0930 | -0.0913 | -0.2157 | 1 |  |
|  | AI | -0.4273 | 0.8088 | -0.4245 | 0.3414 | 0.1664 | 0.6176 | -0.6422 | 0.4321 | 0.4607 | -0.1382 | 0.0747 | -0.2038 | 0.3878 | 1 |

**Table S3**. Mean standardised regression coefficients for biotic and abiotic variables at 0-100% thresholds.

|  | Factors | MAT | MAP | Longitude | Elevation | SWC | pH | w.MNTD | MNTD | CWM.SLA | w.FDis | FDis | Species richness |
| --- | --- | --- | --- | --- | --- | --- | --- | --- | --- | --- | --- | --- | --- |
| Total herbs | Standardized regression coefficients | -0.0613 | 0.0443 | 0.0271 | -0.0001 | 0.1376 | -0.3619 | 0.0010 | 0.0025 | 0.0205 | 0.0072 | 0.2940 | 0.1420 |
|  | Std. Error | 0.0350 | 0.0312 | 0.0255 | 0.0004 | 0.0921 | 0.0496 | 0.0213 | 0.0181 | 0.0108 | 0.0516 | 0.0556 | 0.0965 |
| Perennial herbs | Standardized regression coefficients | -0.0476 | 0.0140 | 0.0742 | -0.0761 | 0.1170 | -0.3970 | 0.0008 | 0.0021 | 0.0343 | 0.3260 | 0.1486 | 0.1471 |
|  | Std. Error | 0.0423 | 0.0413 | 0.0331 | 0.0342 | 0.0917 | 0.0511 | 0.0282 | 0.0232 | 0.0124 | 0.0361 | 0.0469 | 0.0362 |
| Annual herbs | Standardized regression coefficients | -0.0706 | 0.0455 | 0.0345 | -0.0001 | 0.1530 | -0.3340 | 0.0030 | 0.0036 | 0.0240 | 0.0205 | 0.2626 | 0.1680 |
|  | Std. Error | 0.0377 | 0.0332 | 0.0263 | 0.0004 | 0.0968 | 0.0553 | 0.0323 | 0.0486 | 0.0171 | 0.0623 | 0.0621 | 0.1070 |
